# Neutrophils take center stage in VEXAS syndrome pathogenesis

**DOI:** 10.1172/JCI199299

**Published:** 2025-11-03

**Authors:** Ajay Tambralli, Jason S. Knight

**Affiliations:** 1 Division of Rheumatology, Department of Internal Medicine, and; 2 Division of Pediatric Rheumatology, Department of Pediatrics, University of Michigan, Ann Arbor, Michigan, USA.

## Abstract

Vacuoles, E1 enzyme, X-linked, autoinflammatory, somatic (VEXAS) syndrome is an adult-onset inflammatory disorder caused by somatic *UBA1* mutations in hematopoietic stem cells. *UBA1* encodes a key enzyme that catalyzes protein ubiquitination. Clinically, VEXAS is characterized by systemic inflammation and hematologic abnormalities. Patient studies have hinted that the transition of *UBA1*-mutated stem cells into proinflammatory myeloid precursors may propagate the manifestations of VEXAS syndrome. In this issue of the *JCI*, Dong and colleagues developed nine unique conditional knockout mouse strains and found that only neutrophil-specific *Uba1* deletion reproduced VEXAS syndrome–like findings. The observed phenotype was at least in part due to inflammatory reprogramming and longer survival of the mutant neutrophils. In addition to deepening our mechanistic understanding of VEXAS syndrome pathogenesis, this work should provide a platform to pursue more targeted approaches to treatment.

## VEXAS syndrome

Vacuoles, E1 enzyme, X-linked, autoinflammatory, somatic (VEXAS) syndrome is a recently described autoinflammatory disorder caused by somatic mutations in the *UBA1* gene of hematopoietic stem cells (1). *UBA1* is an X-linked gene that encodes the major E1 ubiquitin–activating enzyme. The identification of VEXAS syndrome in 2020 bridged the subspecialties of hematology and rheumatology, illuminating a causal genetic etiology for adult-onset inflammatory disease. Affected individuals are most commonly older men presenting with hematologic abnormalities, such as cytopenias (e.g., macrocytic anemia), vacuoles in erythroid and myeloid precursors, and bone marrow dysplasia. At the same time, systemic inflammation in affected individuals can be particularly severe, affecting multiple organs, including the skin, lungs, vasculature, and cartilaginous structures. Patients with VEXAS syndrome sometimes meet criteria for relapsing polychondritis, polyarteritis nodosa, giant cell arteritis, Sweet syndrome, multiple myeloma, and/or myelodysplastic syndrome, shedding new light on the potential underpinnings of a variety of rheumatologic and hematologic conditions.

Epidemiologic studies suggest that pathogenic and likely pathogenic *UBA1* variants are more common than expected, with prevalence estimates near 1 in 4,000 men older than 50 years, albeit with evidence of incomplete clinical penetrance of VEXAS syndrome–associated *UBA1* variants (2, 3). Treatment approaches for the inflammatory manifestations of VEXAS syndrome have included broad-acting corticosteroids, as well as specific inhibitors of IL-1, IL-6, and JAK/STAT signaling (4, 5). In patients who are refractory to these therapies or in those with severe hematologic manifestations, hypomethylating agents such as azacitidine have been deployed (4, 5). Despite these varied approaches, patients with VEXAS syndrome are prone to incomplete responses to therapy and frequent relapses, with a 12%–40% mortality rate (6–8).

Although considerable progress has been made in defining the clinical phenotypes associated with VEXAS syndrome, fundamental mechanistic questions remain unresolved. Analyses of patient-derived samples have provided valuable insights but fail to fully capture the nuances associated with clonal hematopoiesis, lineage-specific contributions, and cell-cell interactions. Moreover, VEXAS syndrome cells are fragile, have a limited ability to proliferate, and do not tolerate genetic manipulation (9). As a result, there is a lack of understanding of how *UBA1* mutations in hematopoietic stem cells are linked to effector cell dysfunction, inflammation, and bone marrow failure.

## Insights from patients with VEXAS syndrome

Pathogenic and likely pathogenic mutations associated with VEXAS syndrome cluster at the methionine-41 codon of *UBA1*, selectively reducing the expression of the cytoplasmic UBA1b isoform, while sparing nuclear UBA1a (1). Disease severity in VEXAS syndrome appears to be inversely correlated with residual translation of UBA1b (10). Moreover, in patients with VEXAS syndrome, impairment in ubiquitination promotes stress responses in hematopoietic stem and progenitor cells (HSPCs), which positively correlate with inflammation (9). In the bone marrow, *UBA1* mutations associated with VEXAS syndrome can be detected in HSPCs, myeloid progenitors, lymphoid progenitors, and megakaryocytes; however, in the peripheral blood, these mutations are observed only in myeloid cells (1).

Transcriptomic profiling of whole blood, monocytes, and neutrophils has demonstrated gene expression signatures that suggest activation of innate immune pathways, along with increased cell-intrinsic myeloid inflammation (1). Complementary findings are reflected in the peripheral blood cytokine profiles of patients with VEXAS syndrome (1, 11). Single-cell RNA-Seq of VEXAS syndrome bone marrow has suggested that the myeloid bias is an early event, as mutated lymphoid progenitors tend to demonstrate accelerated apoptosis (9). Together, these data support a model in which the clonal expansion of UBA1-mutant hematopoietic stem cells (HSCs) gives rise to dysfunctional, myeloid-skewed progeny that propagate inflammation in VEXAS syndrome. Yet the relative contribution of individual lineages has remained unclear, underscoring the need for a system to establish causality.

## Suggestive evidence implicating neutrophils in VEXAS syndrome pathogenesis

Neutrophils are short-lived cells of myeloid lineage and the most abundant circulating leukocytes in peripheral blood. They respond rapidly to inflammatory cues, survive longer in inflamed tissues, and are sources of tissue-damaging inflammatory mediators, including IL-1β, IL-6, TNF-α, and ROS (12). VEXAS syndrome neutrophils have enriched ROS-related pathways and demonstrate spontaneous neutrophil extracellular trap (NET) formation, which can drive tissue injury and amplify systemic inflammation (1, 9). Furthermore, prominent neutrophil infiltration is observed in skin, lung, and vascular lesions of patients with VEXAS syndrome, reinforcing the likely role of neutrophils in tissue damage (13–15). Together, these observations suggest that neutrophils may be potential effectors of VEXAS syndrome pathogenesis; however, their causal role has not yet been established.

## A lineage-focused approach to *Uba1* knockouts

In this issue of the *JCI*, Dong and colleagues address the challenge of identifying the foundational drivers of VEXAS syndrome manifestations by systematically generating nine conditional knockout mouse strains (16). Using lineage-specific Cre drivers, they ablated *Uba1* in HSCs, lymphoid cell subsets, monocytes/macrophages, megakaryocytes, and neutrophils, thereby directly interrogating which cellular subsets are most closely linked to the autoinflammation observed in VEXAS syndrome.

HSC-specific *Uba1* deletions were not tolerated, resulting in marrow failure and the death of male mice either at the embryonic stage or shortly after Cre induction. Deletion in lymphoid cell subsets and megakaryocytes resulted in lineage-restricted cytopenias, but no systemic inflammation was observed. Monocyte/macrophage-restricted deletion was surprisingly well tolerated, despite the reported monocyte dysregulation in patients with VEXAS syndrome (11). In contrast, neutrophil-specific deletion (via S100a8-Cre) of *Uba1* recapitulated a VEXAS-like syndrome, including neutrophilia, macrocytosis, vacuolated myeloid precursors, splenomegaly, skin (but not cartilage) inflammation, and elevated systemic cytokine levels (IL-1β, IL-6, and TNF-α). This provides critical, direct evidence that neutrophil dysfunction alone can drive at least some features of VEXAS syndrome.

## Neutrophil reprogramming

Dong et al. presented several mechanistic analyses highlighting how *Uba1* deficiency reprograms the biology of neutrophils. Transcriptomics and proteomics profiling revealed loss of ubiquitination signatures, along with induction of the unfolded protein response (UPR), oxidative stress pathways, and inflammatory gene expression modules. Functionally, *Uba1*-deficient neutrophils exhibited increased ROS production, inflammasome activation, and NET formation, alongside high cytokine secretion. These features mirror abnormalities described previously in patient-derived neutrophils (1).

Using competitive bone marrow transplantation and lineage tracing, the authors found that the mutant neutrophils had prolonged survival. Despite flow cytometric profiling that might suggest accelerated apoptosis of mutant neutrophils, these neutrophils actually lived longer, likely supported by cytokine-driven survival signals. This sets up the potential for a feed-forward circuit in which dysfunctional neutrophils amplify systemic inflammation through both cytokine production and a longer lifespan.

## Therapeutic insights: cytokine blockade and neutrophil longevity

The model developed by Dong et al. also allowed the authors to preliminarily investigate the effects of different therapies. IL-1 blockade with either anakinra or canakinumab partially mitigated inflammatory features, reducing leukocytosis and systemic cytokines, in line with the modest efficacy of IL-1 inhibition in patients (4). Interestingly, genetic deletion of *Morrbid*, a long noncoding RNA known to promote myeloid survival, curtailed neutrophil expansion, cytokine production, and systemic inflammation, suggesting that unique neutrophil-targeting strategies could be viable therapeutic options.

## Translational implications and future directions

**Figure 1:**
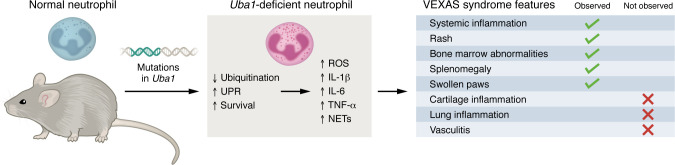
VEXAS syndrome–like findings in mice with neutrophil-specific *Uba1* deficiency. Mouse neutrophils with *Uba1* deficiency exhibited decreased protein ubiquitination, increased UPR, prolonged lifespans, and increased production of ROS, proinflammatory cytokines, and NETs. This mouse model recapitulated some of the clinical features seen in human VEXAS syndrome.

The framework presented by Dong and colleagues highlights a sustainable mouse model to study VEXAS syndrome. It also provides direct evidence that neutrophils are sufficient to drive systemic VEXAS syndrome–like inflammation, linking dysregulated ubiquitination to protein processing failure, prolonged neutrophil survival, and neutrophil inflammatory reprogramming (Figure 1). The work also creates a unique opportunity to evaluate various therapies and their combinations.

While this study has the potential to be transformative, it also has some limitations. A key issue is that the model deletes both UBA1a and UBA1b isoforms, whereas most cases of human VEXAS syndrome arise from missense mutations that selectively downregulate UBA1b, while sparing UBA1a. More broadly, although neutrophils are sufficient to trigger systemic autoinflammation in mice, human VEXAS syndrome is likely a multi-lineage disorder. Clinical manifestations such as cartilage inflammation, pulmonary disease, and marrow failure likely reflect contributions from monocytes, erythroid, and megakaryocytic progeny. Compound lineage perturbations may be necessary to map these interactions. Furthermore, therapeutic responses observed in the null context (e.g., IL-1 blockade, *Morrbid* deletion) may not fully account for the biology of partial loss-of-function alleles. Allele-precise models, focusing specifically on the methionine-41 codon, may be needed to reconcile these differences and faithfully model disease pathogenesis. That said, the model presented by Dong and colleagues here could facilitate in-depth profiling of neutrophil-specific aberrations, which could help define mechanisms underlying their increased longevity and activity, such as escape systems from autophagy, alterations in metabolism, and neutrophil–endothelial cell interactions.

## Conclusion

Dong and colleagues have established a lineage-specific mouse model of VEXAS syndrome and found that neutrophils are at the center of disease pathogenesis. Going forward, this model has the potential to provide a powerful platform for dissecting mechanisms and evaluating therapies, while moving the field closer to personalized treatment strategies.

## Funding support

- Career Development Award, Rheumatology Research Foundation (to AT).

## Version 1 11/03/2025

Electronic publication
